# Colorectal adenocarcinoma with clear cell changes: immunohistological and molecular findings in three cases

**DOI:** 10.1007/s00428-024-03870-0

**Published:** 2024-07-23

**Authors:** Andreas Gocht, Carsten Heidel, Jutta Kirfel, Rita Vesce, Pamela Lazar-Karsten, Helen Pasternack, Madelaine Melzer, Phillip Hildebrand, Nicole Warkentin, Hendrik Schimmelpenning, Verena-Wilbeth Sailer

**Affiliations:** 1 Institut für Pathologie, Universitätsklinikum Schleswig-Holstein, Campus Lübeck, Ratzeburger Allee 160, 23538 Lübeck, Germany; 2 Institut für Pathologie, Universitätsklinikum Düsseldorf, Moorenstr. 5, 40225 Düsseldorf, Germany; 3 Klinik für Allgemein-, Viszeral- und Gefäßchirurgie, Schön Klinik, Neustadt, Am Kiebitzberg 10, 23730 Neustadt in Holstein, Germany

## Introduction

The primary clear cell colorectal adenocarcinoma (CCCRC) is a rare distinctive tumor subtype with until now at least 73 cases reported in the literature (Supplementary Table 1), including three own cases. CCCRC occurs with a frequency between 0.09% and 0.72% of all subtypes of colorectal adenocarcinomas [1, 2]. CCCRC was not included in the current World Health Organization (WHO) 5th classification of tumors of the digestive tract [3]. However, it can be found as a distinct tumor entity in the Armed Forces Institute of Pathology (AFIP) Atlas of Tumor Pathology [4]. CCCRC may express the enteroblastic markers α-fetoprotein (AFP), glypican-3 (GPC3), or spalt-like transcription factor 4 (SALL4) (Supplementary Table 1) [1, 5]. CCCRC is most commonly found in the left colon and men over the age of 40 are more frequently affected than women [2, 4]. The clear cell transformation is not yet fully understood, and the immunohistological and molecular characteristics are inconsistent with the immunohistological positivity of the intestinal markers CK20 and CDX2 as a minimum consensus.

Due to the rarity of well-documented cases, the exact clinical prognosis, pathomorphological and molecular characterizations, and adequate therapeutic strategies of CCCRC are not well known as of now. Here, we present three cases with clinical, pathomorphological, and molecular findings.

### Material and methods

#### Histology

Tissues were formalin-fixed and embedded in paraffin and tissue sections stained with hematoxylin and eosin and PAS. Primary antibodies (Supplementary Table 2) for immunohistology were used as ready-to-use solutions, and stainings were conducted with a Ventana BenchMark Ultra (Ventana/Roche Diagnostics, Mannheim, Germany). The staining results were evaluated as indicated in Table 1.

**Table 1:** Epidemiologic, clinical, immunohistochemical (IHC), and molecular findings

Case	Age/gender	Localization	Macroscopy	Grading/budding^1^	TNM	Therapy	Follow-up	Tumor pattern^2^	CK20	CDX2	SATB2	CEA	MMR	CAIX
No. 1	70 m	Rectum	Polypoid 7,5 cm	G3 (high-grade) CC and CV: 5 Buds (Bd2)	pT2 pN0 (0/16) pM0 L0 V0 Pn0	Ultralow anterior recto-sigmoid resection with total meso-rectal excision	Alive 9 months after surgery, no tumor recurrence	CC (30%) CV (70%)	( +) ( +)	+ + + +	+ + +	( +) + +	+ + + +	( +) ( +)
No. 2	69 m	Rectum, liver	Polypoid 3,5 cm	G3 (high-grade) CC and CV: 6 Buds (Bd2)	pT3 pN2b (8/13) pM1a (HEP) L1 V0 Pn0	Rectum resection Resection solitary liver metastasis Chemotherapy with oxaliplatin, folinic acid, 5-FU	Alive 12 months after therapy, no tumor recurrence	CC (80%) CV (20%)	+ +	+ + + +	─ ( +)	( +) ( +)	+ + + +	( +) ( +)
No. 3	75 m	Ascending colon	Polypoid 5,5 cm	G2 (low-grade) CC: 0 Buds (Bd1) CV: 2 Buds (Bd1)	pT1 (sm3) pN0 (0/21) pM0 L0 V0 Pn0	Right hemicolectomy with MCE	Alive 6 months after therapy, no tumor recurrence	CC (40%) CV (60%)	+ + + +	+ + +	( +) + +	( +) + +	+ + + +	─ ─

Case	PAX8	AFP	GPC3	SALL4	CD10	CgA	Syn	CD56	p53	MUC1	MUC2	MUC5AC	Molecular findings	
No. 1	─ ( +)	( +) ( +)	( +) +	─ ─	+ + + +	( +) ( +)	( +) ( +)	─ ─	+ + + +	( +) ( +)	─ ─	─ ─	CC and CV: *TP53* (c.794 T > C p.L265P (Exon 8)), MSS	
No. 2	─ ─	─ ─	( +) ( +)	─ ( +)	+ + + +	( +) ( +)	( +) ( +)	( +) ( +)	+ + + +	( +) ( +)	( +) ─	─ ─	CC and CV: *TP53* (c.473G > A p.R158H (Exon 5) and c.818G > A p.R273H (Exon 8)), MSS	
No. 3	─ ─	─ ─	─ ─	─ ─	─ ─	─ ─	( +) ( +)	─ ─	+ + + +	─ ─	( +) ─	( +) ─	CC: *TP53* (c.517G > T p.V173L (Exon 5)), *SMAD4* (c.1017 T > A p.F339L (Exon 9)), MSS CV: *TP53* (c.517G > T p.V173L (Exon 5)), *ATM* (c.2546 T > C p.V849A (Exon 17)), MSS	

^1^Tumorbudding per 0.785 mm^2^ (according to ITBCC [3]); ^2^tumor pattern denotes the amount of CC and CV components in percentage; IHC intensities were scored as follows: +  + strong specific staining in ≥ 50% of tumor cells; + faint to moderate specific staining in ≥ 50% of tumor cells; ( +) specific staining in any intensity in < 50% of tumor cells; ─ negative staining. No expression of CK7, RCC, vimentin, and MUC6 in CC and CV; *MMR*, mismatch repair proteins; *CAIX*, carboanhydrase IX; *GPC3*, glypican-3; *CgA*, chromogranin A; *Syn*, synaptophysin

#### DNA extraction and NGS

For each case, tissue areas with preferably high tumor cell content were selected for nucleic acid extractions. Isolation of genomic DNA was performed using the Maxwell RSC DNA FFPE Kit and the Maxwell RSC instrument (Promega, Fitchburg WI, U.S.A.). DNA samples were quantified using the Qubit fluorimeter (Thermo Fisher, Waltham MA, USA). To identify genetic alterations, three cancer panels were used as described in Supplementary Table 2.

## Results

Among approximately 3800 cases with resected colorectal and anal cancers documented in the database of the Department of Pathology at the University of Luebeck, we identified three cases with CCCRC in the years 2001, 2009, and 2023, which corresponds to a frequency of 0.08%. At the time of completing this research, all patients have survived their tumor disease. All relevant data are summarized in Table 1.

**Fig. 1:**
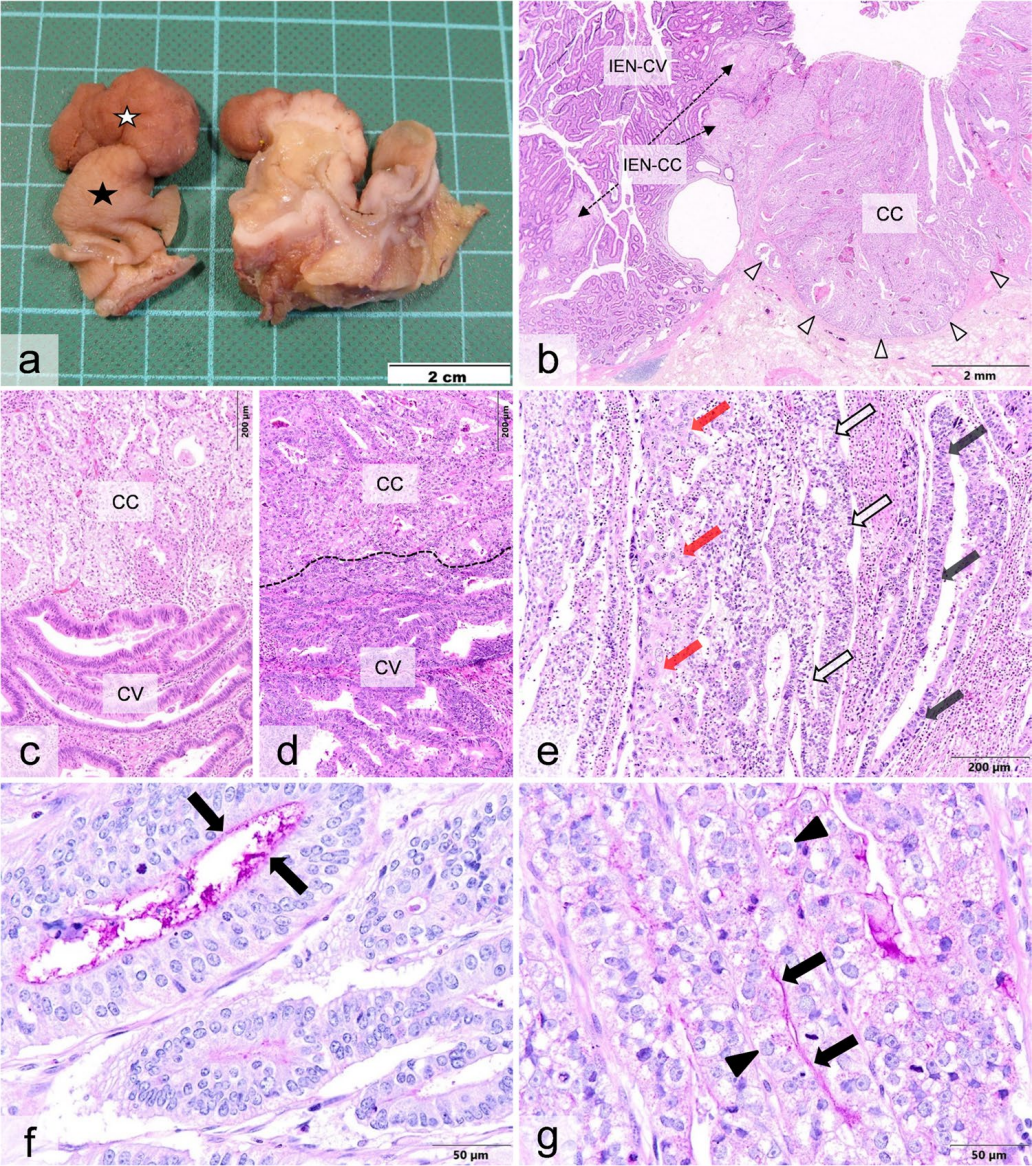
Macroscopical, histo-, and cytomorphological findings in colorectal adenocarcinoma in conventional (CV) and clear cell (CC) components in case 3 (**a, b, c, f, g**), case 1 (**d**), and case 2 (**e**). **a** Macroscopic finding in the formalin-fixed specimen. The polypoid tumor exhibits a homogenous brownish color (white star) on the external surface and a light brown to white color on the cut surface. The unaffected mucosa has a light brown color (black star). **b** Conventional tubulovillous adenoma (IEN-CV) as precursor lesion, which has progressed into a well-differentiated adenocarcinoma (not shown). The adenoma contains foci of tumor glands with clear cell changes (IEN-CC). CC component infiltrates the submucosa. Arrowheads point to invasion front. **c** CV and CC components, which are both low-grade differentiated appear sharply demarcated from each other with pushing margins. **d** Merging of CV and CC components, which are both high-grade differentiated. Dashed line denotes the blurred boundary of both components. **e** Tumor area with different patterns consisting of conventional (light gray arrows), clear cell (white arrows), and undifferentiated hepatoid types (red arrows). **f, g** PAS stains decorating the luminal cell membrane in CV (**f**) and CC component (**g**) marked by arrows. The tumor cells of the CC component are characterized by a lightened cytoplasm and contain depolarized enlarged nuclei with distinct nucleoli. Some clear cells contain fine PAS-positive granular deposits (arrowheads)

**Fig. 2:**
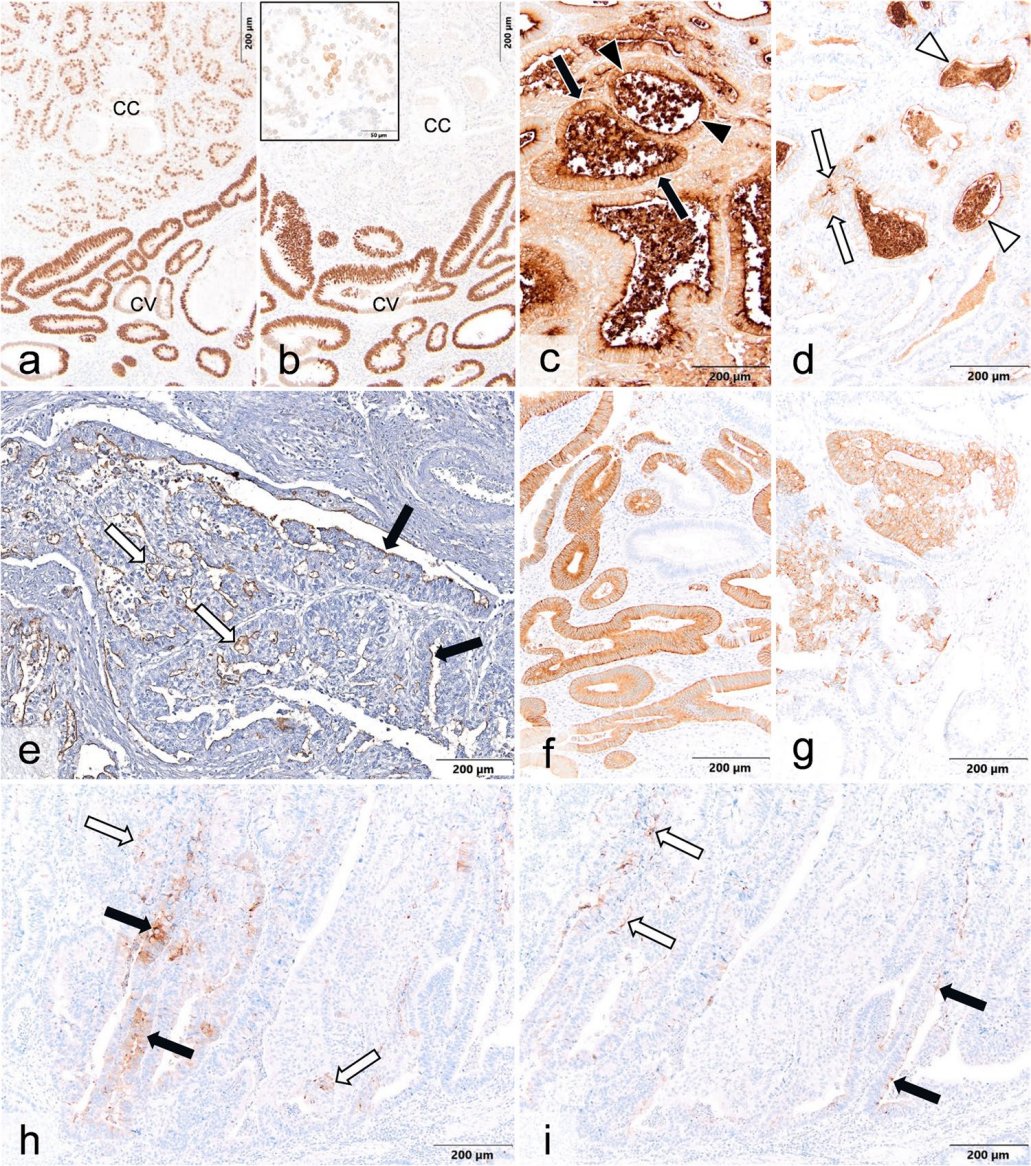
Immunohistological findings in case 3 (**a–d, f, g**), case 2 (**e**), and case 1 (**h, i**). **a** Homogenous nuclear labeling for CDX2 is evident in the conventional (CV) and clear cell (CC) components. Labeling in CC component is less intensive than in CV. **b** Immunostains for SATB2 with strong homogenous labeling of the nuclei of the conventional type only, whereas within the clear cell component, only a few tumor cells show faint nuclear staining (inset). **c, d** For CEA, a strong homogeneous cytoplasmic (black arrows) and membranous (black arrowheads) labeling is seen in the CV component (**c**), whereas the overall staining of the tumor cells in the CC component (**d**) is less intensive (white arrows indicate cytoplasmic and white arrowheads membranous staining). **e** For MUC1, a membranous decoration of tumor cells in the CC component (white arrows) and the CV component (black arrows) is recognizable. **f, g** For synaptophysin, a fine granular cytoplasmic labeling is seen in the CV (**f**) and CC (**g**) components. Immunostains for AFP (**h**) and glypican-3 (**i**) reveal focal faint membranous and cytoplasmic decoration of scattered tumor cells in both CV (black arrows) and CC (white arrows) components

Macroscopically, polyps were present with an external homogenous brownish color and a light brown to white color on the cut surface (Fig. 1a). The histologically distinguishable conventional (CV) and clear cell (CC) tumor components could not be macroscopically differentiated from each other by either color or consistency. In one case (case 3), a tubulovillous adenoma with focal high-grade dysplasia was present as a precursor lesion (Fig. 1b). This was composed of conventional and clear cell glands both with transition to invasive carcinoma. In all three cases, the invasive carcinomas consisted of a mixed pattern of a conventional type and a clear cell component, either sharply demarcated (Fig. 1c) or blended with each other (Fig. 1d), comprising between 30 and 80% of the total tumor area. In the PAS reaction, both tumor components exhibited minimal intra- or extracellular globular deposits and a faint cell surface decoration (Fig. 1f, g). Both tumor patterns were immunohistologically positive for CK20, CDX2, and CEA (Fig. 2a, c, d) with predominantly equal intensities in the cells of the CC and CV components, respectively, whereas SATB2 was significantly reduced in the clear cells (Fig. 2b). Additionally, both tumor components were microsatellite-stable, and a minor component of about 10% revealed neuroendocrine features (Fig. 2f, g). One tumor (case 2) was partially composed of undifferentiated hepatoid tumor cells (Fig. 1e) which failed to immunohistochemically react for any of the enteroblastic markers. In two cases, clear cells were positive for AFP and/or GPC3 (Fig. 2h, i), whereas SALL4 was detected in the conventional part only. The mucin glycoproteins (MUC) showed inconsistent staining results among the individual cases and regarding the clear cell and conventional tumor patterns (Fig. 2e). In the DNA sequencing, both tumor components presented with an identical point mutation in the *TP53* gene. Additional point mutations were detectable in case 3 in the *SMAD4* gene exclusively in the clear cell and in the *ATM* gene only in the conventional component.

## Discussion

The primary CCCRC is defined as a tumor either completely or partially composed of tumor cells with clear cytoplasm similar to clear cell tumors of the lung or genital tract [4]. CCCRC is not recognized as a subtype in the current 5th WHO classification of tumors of the digestive tract, in which clear cell carcinomas are only defined for the stomach, pancreas, bile duct, gall bladder, and liver [6]. Some authors require the presence of more than 50% of the clear cell component to consider the diagnosis of a clear cell adenocarcinoma of the colorectum [7]. However, it must be considered that this cutoff is arbitrary and has not been rigorously evaluated at this point. The mean age of affected individuals is 58.7 years with a median of 59 years (Supplementary Table 1). These patients are younger than those with conventional colorectal adenocarcinoma, which has a median affected age of 70 years at the time of diagnosis [8]. The ratio between female and male is 1:1.8 (Supplementary Table 1). The tumor is most commonly found in the left colon, including the rectum in 72.9% of the reported cases (Supplementary Table 1). Metastases are found in locoregional lymph nodes in 64.3% and in distant regions in 37.1%, affecting the liver in 25.7% (Supplementary Table 1) to 38.7% [5] and the lungs in 11.4% (Supplementary Table 1).

Histologically, CCCRC can present in a pure clear cell or mixed form [4, 7, 9]. The immunohistological marker profile in CCCRC typically shows the expression of colorectal tract antigens, i.e., CK20, CDX2, SATB2, and CEA [2, 7, 10]. Only a small fraction of CCCRC expresses enteroblastic markers with a reported frequency of 5.6% in one series with 303 cases [5]. The expression of enteroblastic markers is a distinctive finding of “colorectal adenocarcinoma with enteroblastic differentiation” (CAED); however, it should be noted that CAED may present with or without clear cell changes [1, 5]. Additional immunohistological markers such as RCC, CD10, carboanhydrase IX, PAX8, and p63 are useful to differentiate CCCRC from other clear cell tumors, e.g., renal cell carcinomas, malignant Müllerian tumors, and squamous cell carcinomas of the anal canal, which all may affect the colorectal tract [2]. The exact cause of clear cell transformation is unknown. The accumulation of glycogen or mucin seems unlikely [11, 12]. Cell clearing may be caused either by degenerative cell processes [9], e.g., lipid-like material [12], or by a transition (metaplasia) of tumor cells from an initially conventional type [11, 12].

In some studies, including one own case, precursor lesions were found, presenting with colorectal clear cell adenomas progressing into invasive CCCRC, however, without yet identifying the underlying molecular pathway [2, 9].

In our cases, mutations in the *TP53* gene (exons 5 and 8) were detectable with identical point mutations in the clear cell and conventional component. Molecular pathological investigations in CCCRC are rare and have revealed several mutations most frequently in the genes *KRAS* and *TP53* (Supplementary Table 1), which are not clear cell-specific as shown in our study and by others [1, 13]. However, somatic mutations in the *TP53* gene are one of the most frequent genetic changes in human cancers overall. Its prevalence in sporadic colorectal carcinomas is 43.2% [14] and affects most frequently the exons 5 to 8 [15]. In one of our cases, we observed a mutation of the *SMAD4* gene in the clear cell component, which has not been previously reported to our knowledge. In gastric adenocarcinoma with enteroblastic differentiation, an inactive *SMAD4* is associated with a worse clinical outcome [16].

Clinically, clear cell colorectal adenocarcinoma appears to have a worse prognosis than conventional subtypes [5, 9]. In one study [1], the 3-year overall survival was only 66% compared to 85% for colorectal adenocarcinomas lacking clear cell differentiation. However, due to the small number of cases, there is no valid data on survival times. Suggested therapeutical strategies are based on single case reports and include HER2-targeted and chemoradiotherapy, followed by surgical removal of the tumor [1, 17]. One of our patients received FOLFOX-6 therapy post-surgery and remained cancer-free for 12 years to date. In contrast, in a 26-year-old female patient, a similar therapy resulted in an insufficient significant clinical improvement [10].

## Conclusion

CCCRC most often appears as a composition of both a conventional and a clear cell component. In accordance with the definition of mucinous colorectal adenocarcinoma, we propose proceeding in a similar manner for clear cell carcinoma, i.e., tumors with histologically proven clear cell differentiation in > 50% of the tumor area should be defined as “clear cell adenocarcinoma” and those with ≤ 50% clear cell changes as “adenocarcinoma with clear cell features.” The clear cells may express AFP, glypican-3, and SALL4. However, these enteroblastic markers should not be a prerequisite for the diagnosis as these antigens are only inconsistently detectable.

## Supplementary Information

Below is the link to the electronic supplementary material.

Supplementary file1 (PDF 381 KB)

Supplementary file2 (PDF 307 KB)
