# Supplementary material for: Colorectal adenocarcinoma with clear cell changes: immunohistological and molecular findings in three cases
Source: Virchows Arch. 2024 Jul 23;485(3):569–74. doi: 10.1007/s00428-024-03870-0 (PMC11415476; doi:10.1007/s00428-024-03870-0)
Supplement: Supplementary file 1 — Supplementary file1 (PDF 381 KB) [file 428_2024_3870_MOESM1_ESM.pdf]

Colorectal adenocarcinoma with clear cell changes. Immunohistological and molecular findings in three cases.
Virchows Archiv.
Andreas Gocht*, Carsten Heidel, Jutta Kirfel, Rita Vesce, Pamela Lazar-Karsten, Helen Pasternack, Madelaine Melzer, Phillip Hildebrand, Nicole Warkentin, Hendrik Schimmelpenning, Verena-Wilbeth Sailer
*Corresponding author: Institut für Pathologie, Universitätsklinikum Schleswig-Holstein, Campus Lübeck, Ratzeburger Allee 160, D-23538 Lübeck, Germany; E-Mail address: Andreas.Gocht@uksh.de

Supplementary Table 1: Cases with colorectal and anal carcinomas with clear cell changes reported in the literature and present report (n=73). Epidemiological and pathological findings

**Author (list of references see below)**	**Age/ Gender**	**Tumor localization**	**Metastases** Loco-regional lymph nodes	Distant	**Enteroblastic markers (AFP/ GPC3/SALL4) positive/negative/not done**	**Mutations in clear cell (CC) and conventional (CV) compartment**
Hellstrom and Fisher 1964	67 m	Rectum	Yes	No	N/A	N/A
Reed et al. 1983	71 m	Transverse colon	N/A	N/A	N/A	N/A
Jewell et al. 1988	75 m<br>56 f	Rectum<br>Left colon	No	No	N/A<br>N/A	N/A
Watson 1990	53 m	Anal canal	Yes	No	N/A	N/A
Hitti et al. 1990	39 f	Sigmoid	No	No	N/A	N/A
Rubio 1995	68 m	Left colon	Yes	Liver, lung	N/A	N/A
Sasaki et al. 1996	49 f	Rectum	N/A	N/A	N/A	N/A
Young and Hart 1998	33 f<br>33 f<br>49 f<br>71 f	Transverse colon<br>Left colon<br>N/A<br>N/A	N/A<br>N/A<br>N/A<br>N/A	N/A<br>N/A<br>N/A<br>N/A	N/A<br>N/A<br>N/A<br>N/A	N/A<br>N/A<br>N/A<br>N/A
Furman and Lauwers 1999	N/A	Rectum	N/A	N/A	N/A	N/A
McCluggage et al. 2001	65 f	Rectum	Yes	Liver, lung, peritoneum, both adrenal glands, para-aortic lymph nodes	N/A	N/A
Braumann et al. 2004	89 m	Left colon	No	No	N/A	N/A
Mallik and Katchy 2005	36 f	Rectum	Yes	No	N/A	N/A
Ko et al. 2007	62 m	Left colon (intramucosal carcinoma)	not defined	not defined	N/A	N/A
Hao et al. 2007	37 m	Descending colon	Yes	N/A	N/A	N/A
Sawai 2007	56 f	Rectum	N/A	N/A	N/A	N/A
Honma 2007	50 f	Rectum	N/A	N/A	N/A	N/A
Barisella et al. 2008	54 m	Right colon	No	N/A	N/A	CC: *KRAS* p.G12V
Bressenot et al. 2008	84 f	Left colon	No	No	N/A	N/A
Pusztaszeri and Bouzourene 2008	62 m	Sigmoid	N/A	N/A	N/A	N/A
Soga et al. 2008	71 f	Right colon (intramucosal carcinoma)	not defined	not defined	N/A	N/A
Eloy et al. 2009	48 f<br>84 m	Transverse Colon (intramucosal carcinoma)<br>Sigmoid (intramucosal carcinoma)	not defined<br>not defined	not defined<br>not defined	N/A<br>N/A	N/A<br>N/A
Finkelstein et al. 2010	41 f	Rectum	No	No		N/A

Shi et al. 2010	52 m	Rectum	N/A	N/A	N/A	N/A
	51 m	Sigmoid	N/A	N/A	N/A	N/A
	61 m	Sigmoid (intramucosal carcinoma)	not defined	not defined	N/A	N/A
	63 m	Rectum (intramucosal carcinoma)	not defined	not defined	N/A	N/A
	35 m	Sigmoid (intramucosal carcinoma)	not defined	not defined	N/A	N/A
Furuya et al. 2011	81 m	Ascending colon	Yes	Liver, lung, peritoneum	AFP+, GPC3N/A, SALL4N/A	N/A
Bakshi et al. 2012	42 m	Transverse colon/hepatic flexure	No	N/A	N/A	N/A
Kanstrup Fiehn et al. 2012	69 m	Sigmoid	Yes	No	N/A	CC: *KRAS* wildtype, MSS
Kyueng-whan 2013	50 f	Rectum	No	No	N/A	N/A
Min et al. 2013	50 f	Rectum	No	No	N/A	N/A
Barrera-Maldonado et al. 2014	41 f	Descending colon	Yes	No	N/A	N/A
Gurzu et al. 2014	82 m	Rectum	Yes	Liver	N/A	CC: *KRAS* codon 12 and 13
Thelin et al. 2014	25 m	Ascending colon/hepatic flexure	Yes	No	AFP+, GPC3N/A, SALL4N/A	N/A
Wang et al. 2014	26 m	Transverse colon/spleen flexure	N/A	No	N/A	N/A
Mhamdi et al. 2017	52 m	Sigmoid	Yes	No	N/A	N/A
Remo et al. 2017	58 m	Right colon	Yes	No	N/A	CC: *BRAF* V600E, *KRAS* wildtype, MSI-H
	79 m	Right colon	No	No	N/A	CC *KRAS* p.G12V, *BRAF* wild-type, MSS
Nakamura et al. 2018	68 m	Rectum	No	No	AFP+, GPC3+, SALL4+	N/A
Tochio et al. 2018	48 m	Descending colon (intramucosal carcinoma)	not defined	not defined	AFP–, GPC3–, SALL4–	N/A
Oyama et al. 2019	58 m	Sigmoid	N/A	No	AFP–, GPC3–, SALL4N/A	N/A
Murakami et al. 2020	52 f	Rectum	No	Lung	AFP+, GPC3+, SALL4+	N/A
	74 m	Sigmoid	Yes	No	AFP–, GPC3+, SALL4+	N/A
	59 m	Rectum	Yes	Liver	AFP–, GPC3+, SALL4–	N/A
	62 m	Sigmoid	Yes	Liver	AFP–, GPC3+, SALL4+	N/A
	84 f	Sigmoid	Yes	No	AFP–, GPC3–, SALL4+	N/A
Shiratori et al. 2020	80 m	Transverse colon (intramucosal carcinoma)	not defined	not defined	AFP–, GPC3+, SALL4–	N/A
Yamashiro et al. 2020 10 cases	x̄ 66.7 m (6) f (4)	Left colon (7) Right colon (3)	Yes (6 cases)	N/A	all cases with at least one marker positive	9 cases with *TP53* (CC and CV not differentiated) p.Pro152Leu c.455C>T and HER2+ (IHC 3 +) p.Val157Phe c.469G>T p.Arg273Cys c.817C>T p.Arg282Trp c.844C>T p.Ile195Thr c.584T>C p.Arg196Ter c.586C>T p.Arg273Cys c.817C>T and p.Ala161Thr c.481G>A p.Tyr163Cys c.488A>G p.Ser241Phe c.722C>T p.Val173Ala c.518T>C

Tochio et al. 2021	57 m	Transverse colon	N/A	N/A	AFP–, GPC3–, SALL4–	N/A
Abada et al. 2022	26 f	Sigmoid	N/A	Liver	AFP+, GPC3N/A, SALL4+	CC: *TP53* p.R175H, *KRAS* mutated (not specified), *MUTYH* p.G396D (exon 13), *KDM6A* p.S532fs (exon 16)
Giuseppe et al. 2022	71 m	Sigmoid	Yes	Liver	AFP+, GPC3+, SALL4+	CC*: KRAS*, *NRAS*, *BRAF* wildtype, MSS, HER2 overexpression (immuno-histologically)
Karačić et al. 2022	65 m	Sigmoid	Yes	N/A	AFP–, GPC3N/A, SALL4N/A	N/A
Khefacha et al. 2022	75 f	Right Colon	No	No	N/A	N/A
Kihara et a. 2022	53 m	Rectum	Yes	No	AFP–, GPC3+, SALL4+	N/A
Li et al. 2023	65 m	Transverse colon	N/A	N/A	AFP+, GPC3N/A, SALL4N/A	N/A
Present study	70 m	Rectum	No	No	AFP+, GPC3+. SALL4–	CC and CV: *TP53* p.L265P, MSS
	69 m	Rectum	Yes	Liver	AFP–, GPC3+, SALL4–	CC and CV: *TP53* p.R158H, *TP53* p.R273H, MSS
	75 m	Ascending colon	No	No	AFP–, GPC3–, SALL4–	CC: *TP53* p.V173L, *SMAD4* p.F339L, MSS<br>CV: *TP53* p.V173L, *ATM* p.V849A, MSS

Abbreviations: m male; f female; N/A not available; GPC3 glypican-3

References:

1. Hellstrom HR, Fisher ER (1964) Physaliferous variant of carcinoma of colon. Cancer 17:259-263. https://doi.org/10.1002/1097-0142(196402)17:2<259::aid-cncr2820170217>3.0.co;2-s

2. Reed RJ, Love GL, Harkin JC (1983) Consultation case. Am J Surg Pathol 7(6):597-601

3. Jewell LD, Barr JR, McCaughey WT, Nguyen GK, Owen DA (1988) Clear-cell epithelial neoplasms of the large intestine. Arch Pathol Lab Med 112(2):197-199

4. Watson PH (1990) Clear-cell carcinoma of the anal canal: a variant of anal transitional zone carcinoma. Hum Pathol 21(3):350-352. doi:10.1016/0046-8177(90)90237-y

5. Hitti IF, Glasberg SS, Lubicz S (1990) Clear cell carcinoma arising in extraovarian endometriosis: report of three cases and review of the literature. Gynecol Oncol 39(3):314-320. doi:10.1016/0090-8258(90)90259-n

6. Rubio CA (1995) Clear cell adenocarcinoma of the colon. J Clin Pathol (12):1142-1144. doi:10.1136/jcp.48.12.1142

7. Sasaki M, Miyoshi N, Hirata T, Maruyama H, Nakai H, Sasaki N, et al. (1996) A case of malignant mixed epithelial tumor arising from endometriosis of the rectovaginal septum. J Jpn Pract Surg Soc 57(9): 2268–72

8. Young RH, Hart WR (1998) Metastatic intestinal carcinomas simulating primary ovarian clear cell carcinoma and secretory endometrioid carcinoma: a clinicopathologic and immunohistochemical study of five cases. Am J Surg Pathol 22(7):805-815. doi:10.1097/00000478-199807000-00003

9. Furman J, Lauwers GY (1999) Clear cell change in colonic adenocarcinoma: an equally unusual finding [published correction appears in Histopathology 2000 Feb;36(2):191]. Histopathology 35(5):476-477. doi:10.1046/j.1365-2559.1999.035005476.x

10. McCluggage WG, Desai V, Toner PG, Calvert CH (2001) Clear cell adenocarcinoma of the colon arising in endometriosis: a rare variant of primary colonic adenocarcinoma. J Clin Pathol 54(1):76-77. doi:10.1136/jcp.54.1.76

11. Braumann C, Schwabe M, Ordemann J, Jacobi CA (2004) The clear cell adenocarcinoma of the colon: case report and review of the literature. Int J Colorectal Dis 19(3):264-267. doi:10.1007/s00384-003-0545-5

12. Mallik AA, Katchy KC (2005) Clear cell adenocarcinoma of the rectum. Med Princ Pract 14(1):58-60. doi:10.1159/000081926

13. Ko YT, Baik SH, Kim SH, Min BS, Kim NK, Cho CH, Lee S K, Kim HG (2007) Clear cell adenocarcinoma of the sigmoid colon. Int J Colorectal Dis 22(12):1543-1544. doi:10.1007/s00384-006-0257-8

14. Hao LS, Zhu X, Zhao LH, Qian K, Zhou Y, Bu J, Wu XT (2007) Clear cell adenocarcinoma of colorectum: a case report and review of the literature. Acta Gastroenterol Belg 70(2):235-238

15. Sawai K (2008) Endometrioid adenocarcinoma arising from enddometriosis of the rectum. J Jpn Surg Assoc 69(8): 2063–7. https: //doi.org/10.3919/jjsa.69.2063.

16. Honma S (2009) A case of clear cell adenocarcinoma arising in the endometriosis of the rectum. J Jpn Soc Coloproctol 62:416–419

17. Barisella M, Lampis A, Perrone F, Carbone A (2008) Clear cell adenocarcinoma of the colon is a unique morphological variant of intestinal carcinoma: case report with molecular analysis. World J Gastroenterol 14(42):6575-6577. https://doi.org/10.3748/wjg.14.6575

18. Bressenot A, Marcon N, Montagne K, Plénat F(2008) Clear cell adenocarcinoma: a rare variant of primary colonic tumour. Int J Colorectal Dis 23(1): 137–138

19. Pusztaszeri M, Bouzourene H (2008) Invasive carcinoma arising from a colonic adenoma with clear cell change. Hum Pathol 39(9):1402–1405. https://doi.org/10.1016/j.humpath.2008.01.008

20. Soga K, Konishi H, Tatsumi N, Konishi C, Nakano K, Wakabayashi N, Mitsufuji S, Kataoka K, Okanoue T, Mukaisho K, Hattori T (2008) Clear cell adenocarcinoma of the colon: a case report and review of literature. World J Gastroenterol 14(7):1137-1140. doi:10.3748/wjg.14.1137

21. Eloy C, Lopes JM, Faria G, Moreira H, Brandão A, Silva T, Carneiro F (2009) Clear cell change in colonic polyps. Int J Surg Pathol 17(6):438-443. https://doi.org/10.1177/1066896908319211

22. Finkelstein A, Levy GH, Hui P (2010) Clear cell adenocarcinoma of the colon arising in the background of endometriosis: a case report and diagnostic approach. Lab Med 41(2): 111–114

23. Shi C, Scudiere JR, Cornish TC, Lam-Himlin D, Park JY, Fox MR, Montgomery EA (2010) Clear cell change in colonic tubular adenoma and corresponding colonic clear cell adenocarcinoma is associated with an altered mucin core protein profile. Am J Surg Pathol 34(9):1344-50. https://doi.org/10.1097/PAS.0b013e3181ec0810.

24. Furuya Y, Wakahara T, Akimoto H, Kishimoto T, Hiroshima K, Yanagie H, Yasuhara H (2011) Clear cell adenocarcinoma with enteroblastic differentiation of the ascending colon. J Clin Oncol 29(22):e647-9. https://doi.org/10.1200/JCO.2011.34.9670

25. Bakshi N, Sharma S, Kaushal V, Mardi K (2012) Clear cell adenocarcioma of the colon is a unique morphologic variant of intestinal carcinoma: a rare case report. Clin Cancer Investig J 1(3):173–175

26. Kanstrup Fiehn AM, Andersson S, Grupe Larsen L (2012) Primært clear-celle-adenokarcinom i colon [Primary clear cell adenocarcinoma of the colon]. Ugeskrift for laeger 174(39):2307–2308

27. Kyueng-whan M, Koh YW, Ryu YJ, Hong SM, Kim KR, Sung CO (2013) Primary clear cell adenocarcinoma arising from rectal endometriosis. Dig Endosc 25: 204–215

28. Min K-W, Koh YW, Ryu Y-J (2013) Primary clear cell adenocarcinoma arising from rectal endometriosis. Dig Endosc 25(2): 209–10. https: //doi.org/10.1111/den.12008

29. Barrera-Maldonado CD, Wiener I, Sim S (2014) Clear cell adenocarcinoma of the colon: a case report and review of the literature. Case Rep Oncol Med 2014:905478. https://doi.org/10.1155/2014/905478

30. Gurzu S, Jung I, Bara T, Bara T Jr, Serester O (2014) Immunohistochemical and molecular features of primary clear cell-adenocarcinoma of the rectum, as predictive factors for individualized therapy. Rom J Morphol Embryol 55(2 Suppl):629-633. PMID: 25178336

31. Okazawa Y, Takahashi R, Mizukoshi K, Takehara K, Ishiyama S, Sugimoto K, Takahashi M, Kojima Y, Goto M, Okuzawa A, Tomiki Y, Yao T, Sakamoto K (2014) A case of clear cell adenocarcinoma arising from endometriosis of the rectum treated by laparoscopic surgery. Int J Surg Case Rep 5(12):979-983. doi:10.1016/j.ijscr.2014.10.034

32. Thelin C, Alquist CR, Engel LS, Dewenter T (2014) Primary clear cell adenocarcinoma of the colon: a case report and review. J La State Med Soc 166(4): 143–148

33. Wang W, Li X, Qu G, Leng T, Geng J (2014) Primary clear cell adenocarcinoma of the colon presenting as a huge extracolic mass: a case report. Oncol Lett 8(4): 1873–1875

34. Mhamdi N, Bdioui A, Abdessayed N, Mestiri S, Guerfela, M, Mokni M (2017) Clear cell adenocarcinoma of the colon in the setting of attenuated familial polyposis: a rare variant of primary colonic adenocarcinoma. Next Generat Sequenc Applic 4(1): 1000143. https://doi.org/10.4172/2469-9853.1000143

35. Remo A, Grillo F, Mastracci L, Fassan M, Sina S, Zanella C, Parcesepe P, Damiano Urso E, Pancione M, Bortuzzo G, Scarpa A, Manfrin E (2017) Clear cell colorectal carcinoma: Time to clarify diagnosis. Pathol Res Pract 213(5):447-452. doi:10.1016/j.prp.2017.02.013

36. Nakamura Y, Matsuda K, Yokoyama S, Tamura K, Mitani Y, Iwamoto H, Mizumoto Y, Murakami D, Fujimoto M, Yamaue H (2018) Alpha-fetoprotein-producing rectal cancer successfully responded to preoperative chemoradiotherapy: case report. Surg Case Rep 4(1):111. https://doi.org/10.1186/s40792-018-0520-6

37. Tochio T, Mukai K, Baba Y, Asakawa H, Nose K, Tsuruga S, Kumazawa H, Isono Y, Tanaka H, Matsusaki S, Sase T, Saito T, Okano H, Usui M, Watanabe G (2018) Early stage clear cell adenocarcinoma of the colon examined in detail with image-enhanced endoscopy: a case report. Clin J Gastroenterol 11(6):465-469. doi:10.1007/s12328-018-0889-6

38. Oyama Y, Nishida H, Kusaba T, Kadowaki H, Arakane M, Okamoto K, Wada J, Urabe S, Daa T (2019) Colon adenoma and adenocarcinoma with clear cell components - two case reports. Diagn Pathol 14(1):37. https://doi.org/10.1186/s13000-019-0819-z

39. Murakami T, Yao T, Yatagai N, Yamashiro Y, Saito T, Sakamoto N, Nagahara A (2020) Colorectal adenocarcinoma with enteroblastic differentiation: a clinicopathological study of five cases. Histopathology 76(2):325-332. https://doi.org/10.1111/his.13973

40. Shiratori Y, Suzuki K, Ikeya T (2020) Colonic clear cell adenocarcinoma with enteroblastic differentiation. Clin J Gastroenterol 13(6):1196-1199. https://doi.org/10.1007/s12328-020-01209-1

41. Tochio T, Baba Y, Asakawa H, Nose K, Tsuruga S, Kumazawa H, Isono Y, Tanaka H, Matsusaki S, Sase T, Saito T, Okano H, Mukai K, Kushima R (2021) Early stage clear cell adenocarcinoma coexisting with tubular adenoma and adenoma with clear cell change in the colon. Clin J Gastroenterol 14(2):589-593. https://doi.org/10.1007/s12328-020-01334-x

42. Abada E, Anaya IC, Abada O, Lebbos A, Beydoun R (2022) Colorectal adenocarcinoma with enteroblastic differentiation: diagnostic challenges of a rare case encountered in clinical practice. J Pathol Transl Med 56(2):97-102. https://doi.org/10.4132/jptm.2021.10.28

43. Giuseppe L, Giovanna S, Massimo M (2022) Novel recognition of HER2 amplification in enteroblastic colonic adenocarcinoma. Diagnostic clues and future perspectives. J Surg Res 5(3): 559–562. https://doi.org/10.26502/jsr.10020256

44. Karačić A, Stanić G, Štritof D, Bakula B, Jandrić D, Kekez I (2022) Mucinous adenocarcinoma of the colon with clear cell component: a case report and literature review. Case Rep Pathol 2022:7631686. https://doi.org/10.1155/2022/7631686

45. Khefacha F, Triki W, Ayed K, Abbassi I, Baccar A, Baraket O, Bouchoucha S (2022) Right colon clear cell carcinoma of Müllerian type. Case Rep Gastroenterol 16(2):469-479. https://doi.org/10.1159/000525071

46. Kihara T, Kuwahara R, Kusunoki K, Minagawa T, Horio Y, Uchino M, Ikeuchi H, Hirota S (2022) Rectal carcinoma with dual differentiation toward enteroblastic and neuroendocrine features arising in a patient with ulcerative colitis: a case report. World J Surg Oncol 20(1):371. https://doi.org/10.1186/s12957-022-02838-1

47. Kurosawa T, Murakami T, Yamashiro Y, Terukina H, Hayashi T, Saito T, Nojiri S, Sakamoto K, Nagahara A, Yao T (2022) Mucin phenotypes and clinicopathological features of colorectal adenocarcinomas: Correlation with colorectal adenocarcinoma with enteroblastic differentiation. Pathol Res Pract 232:153840. https://doi.org/10.1016/j.prp.2022.153840

48. Li F, Chen D, Chen J, Nie L (2023) Colonic de novo clear cell adenocarcinoma of intestinal-type: a case report and review of the literature. Int J Surg Pathol 10668969231214808. https://doi.org/10.1177/10668969231214808
