# Supplementary material for: Colorectal adenocarcinoma with clear cell changes: immunohistological and molecular findings in three cases
Source: Virchows Arch. 2024 Jul 23;485(3):569–74. doi: 10.1007/s00428-024-03870-0 (PMC11415476; doi:10.1007/s00428-024-03870-0)
Supplement: Supplementary file 2 — Supplementary file2 (PDF 307 KB) [file 428_2024_3870_MOESM2_ESM.pdf]

Colorectal adenocarcinoma with clear cell changes. Immunohistological and molecular findings in three cases.
Virchows Archiv.

Andreas Gocht*, Carsten Heidel, Jutta Kirfel, Rita Vesce, Pamela Lazar-Karsten, Helen Pasternack, Madelaine Melzer, Phillip Hildebrand, Nicole Warkentin, Hendrik Schimmelpenning, Verena-Wilbeth Sailer
*Corresponding author: Institut für Pathologie, Universitätsklinikum Schleswig-Holstein, Campus Lübeck, Ratzeburger Allee 160, D-23538 Lübeck, Germany; E-Mail address: Andreas.Gocht@uksh.de

Supplementary Table 2: Immunohistochemistry and molecular analysis.

Antibody specification and manufacturer used for immunohistochemistry

Antigen[a]	Clone	Manufacturer	Pretreatment (minutes)
CK20	SP33	Ventana/Roche	CC1 (64 min.)
CDX2	EPR2764Y	Cell Marque	CC1 (32 min.)
SATB2	EP281	Cell Marque	CC1 (32 min.)
CEA	CEA31	Cell Marque	Protease1 (min.)
CK7	SP52	Ventana/Roche	CC1 (24 min.)
CAIX	EP161	Cell Marque	CC1 (32 min.) and Protease3 (4 min.)
PAX8	MRQ-50	Cell Marque	CC1 (64 min.)
RCC	PN-15	Cell Marque	Protease1 (4 min.)
AFP	Polyclonal	Cell Marque	CC1 24 (min.)
GPC3	GC33	Ventana/Roche	CC1 (24 min.)
SALL4	6E3	Cell Marque	CC1 (32 min.)
Vimentin	V9	Ventana/Roche	CC1 (8 min.)
CD10	SP67	Ventana/Roche	CC1 (64 min.) and amplifier
p53	DO-7	Ventana/Roche	CC1 (48 min.)
Chromogranin A	LK2H10	Ventana/Roche	CC1 (8 min.)
Synaptophysin	SP11	Ventana/Roche	CC1 (40 min.)
CD56	MRQ-42	Cell Marque	CC1 (40 min.)
MLH1*	M1	Ventana/Roche	CC1 (48 min.) and amplifier
PMS2*	EPR3947	Cell Marque	CC1 (72 min.) and amplifier
MSH2*	G219-1129	Cell Marque	CC1 (64 min.)
MSH6*	44	Cell Marque	CC1 (72 min.) and amplifier
MUC1	DCM-17	DCS	CC1 (20 min.)
MUC2	CCP58	Agilent	CC1 (40 min.)
MUC5AC	CLH2	Chemicon	CC1 (40 min.)
MUC6	DCM-20	DCS	CC1 (40 min.)

[a]CAIX = Carboanhydrase IX; RCC = Renal cell carcinoma marker; GPC3 = glypican-3; MMR = mismatch repair proteins

Molecular Analysis: To identify genetic alterations, the Ion AmpliSeq™ Colon and Lung Cancer Research Panel v2, the Ion AmpliSeq™ Cancer Hotspot Panel v2, and the Ion GeneStudio™ S5 Systems (all from ThermoFisher Scientific, Carlsbad, CA, U.S.A.) were used. These panels are designed to cover approximately 2,800 COSMIC mutations of oncogenes and tumor suppressor genes. These include *ABL1, AKT1, ALK, APC, ATM, BRAF, CDH1, CDKN2A, CSF1R, CTNNB1, DDR2, EGFR, ERBB2, ERBB4, EZH2, FBXW7, FGFR1, FGFR2, FGFR3, FLT3, GNA11, GNAQ, GNAS, HNF1A, HRAS, IDH1, IDH2, JAK2, JAK3, KDR, KIT, KRAS, MET, MLH1, MPL, NOTCH1, NPM1, NRAS, PDGFRA, PIK3CA, PTEN, PTPN11, RB1, RET, SMAD4, SMARCB1, SMO, SRC, STK11, TP53, VHL.*
